# Supplementary material for: Transparent Cross-Flow Platform as Chemiluminescence Detection Cell in Cross Injection Analysis
Source: Molecules. 2023 Jan 30;28(3):1316. doi: 10.3390/molecules28031316 (PMC9919639; doi:10.3390/molecules28031316)
Supplement: Supplementary file 1 [file molecules-28-01316-s001.zip › molecules-2101645-supplementary.pdf]

Supplementary Material

Transparent Cross-Flow Platform as Chemiluminescence Detection Cell in Cross Injection Analysis

Thachkorn Somboonsuk [1,2], Phoonthawee Saetear [1,2], Thitirat Mantim [1,3], Nuanlaor Ratanawimarnwong [1,3], Prapin Wilairat [1,2], Nathawut Choengchan [1,4,*] and Duangjai Nacapricha [1,2,*]

[1] Flow Innovation-Research for Science and Technology Laboratories (Firstlabs), Ratchathewi District, Bangkok 10110, Thailand
[2] Department of Chemistry and Center of Excellence for Innovation in Chemistry, Faculty of Science, Mahidol University, Rama 6 Road, Ratchathewi District, Bangkok 10400, Thailand
[3] Department of Chemistry, Faculty of Science, Srinakharinwirot University, Sukhumvit 23, Watthana District, Bangkok 10110, Thailand
[4] Department of Chemistry and the Applied Analytical Chemistry Research Unit, Faculty of Science, King Mongkut's Institute of Technology Ladkrabang, Ladkrabang District, Bangkok 10520, Thailand
* Correspondence: nchoengchan@gmail.com or nathawut.ch@kmitl.ac.th (N.C.); dnacapricha@gmail.com or duangjai.nac@mahidol.ac.th (D.N.); Fax: +66-23298428 (N.C.); +66-22015127 (D.N.)

This Supplementary information contains seven figures (Figures S1 to S7) and two tables (Tables S1 to S2)

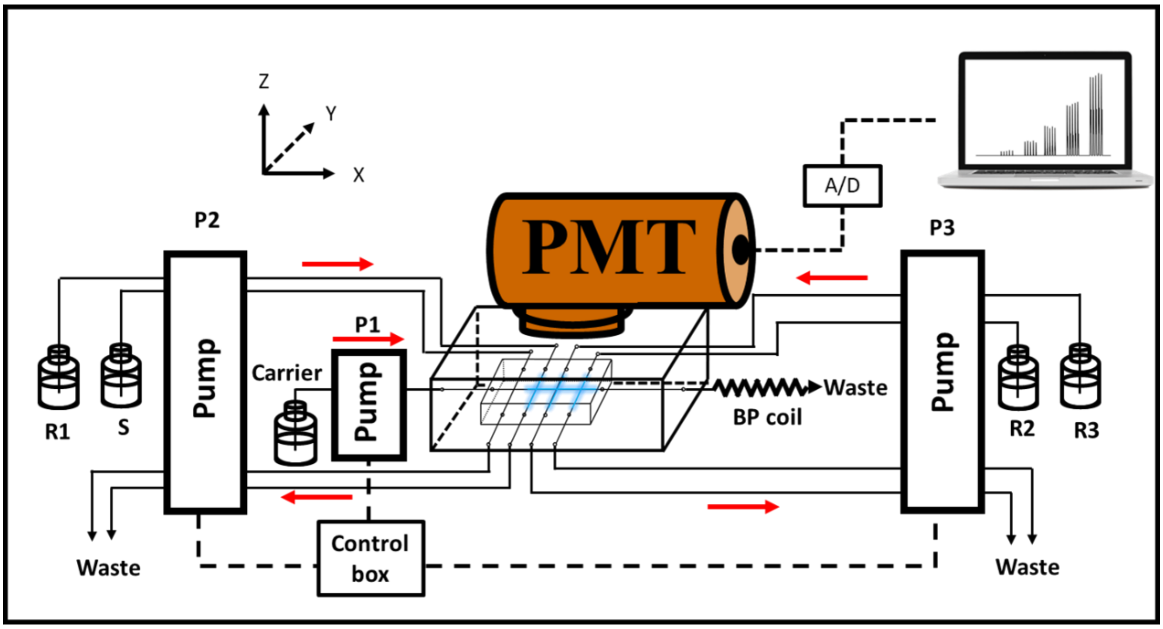

Figure S1. CIA System I for the determination of Co(II). P1, P2, P3 are peristaltic pumps. BP coil: PTFE tube (0.75 mm. i.d., 1,000 mm). A/D: analog to digital converter. C: carrier solution (0.5% (*w*/*w*) H_2O_2 in 0.1 mol L^{-1} carbonate buffer pH 10.5. S: standard Co(II) or sample. R1: 2.5 mmol L^{-1} luminol in 0.05 mol L^{-1} NaOH. R2 and R3 are the same solution as the carrier.

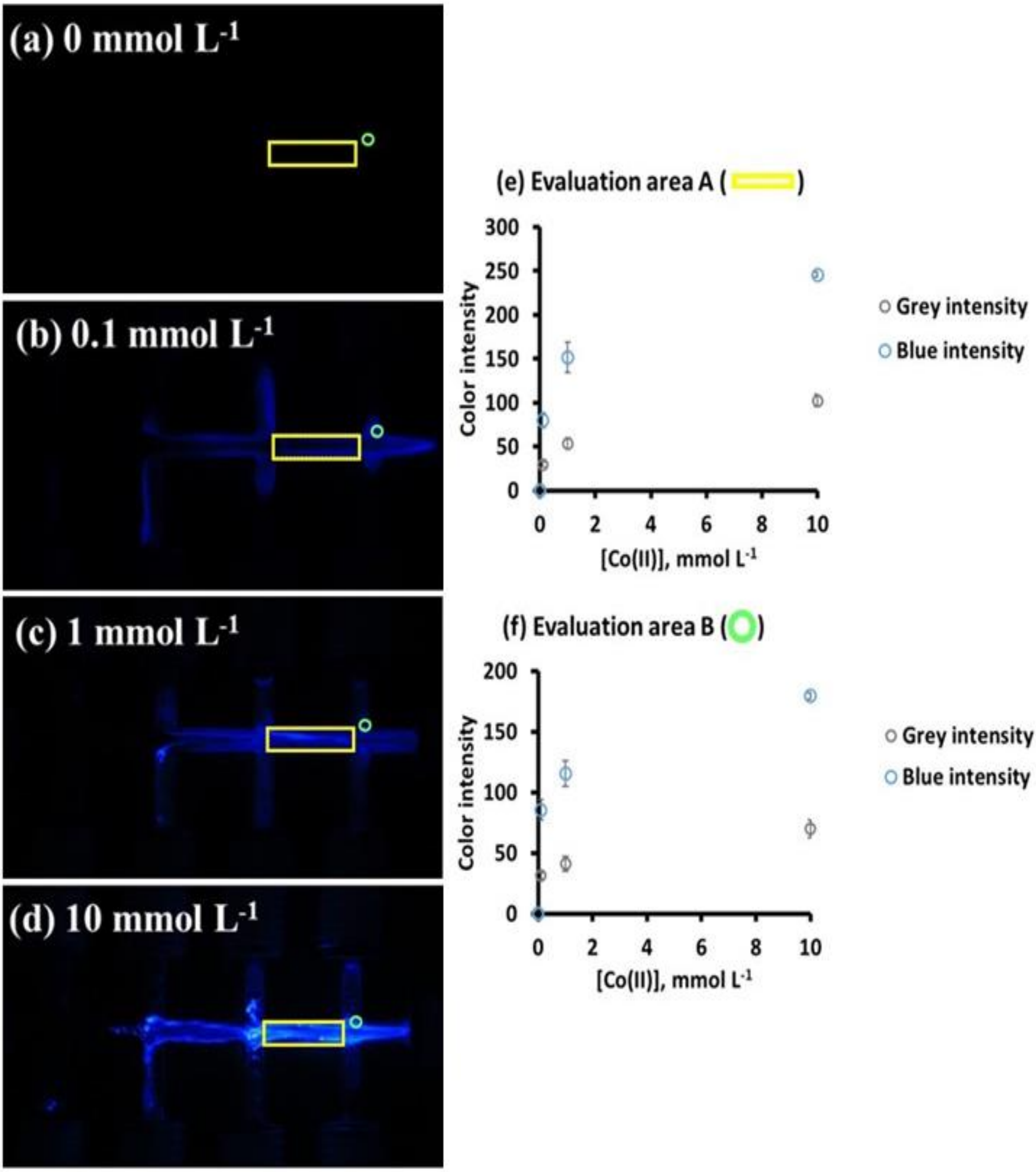

Figure S2. Digital images of the CIA conduit taken at 10 s after aspirating a solution containing Co(II) at 0 to 10 mmol L^{-1} ((**a**) to (**d**), respectively) into the CIA manifold in Figure S1, together with the plot of mean Grey and Blue intensity (from ImageJ program) and concentration of Co(II). Note: The color intensity is measured at two different areas of the conduit: (**e**) area A and (**f**) area B, respectively.

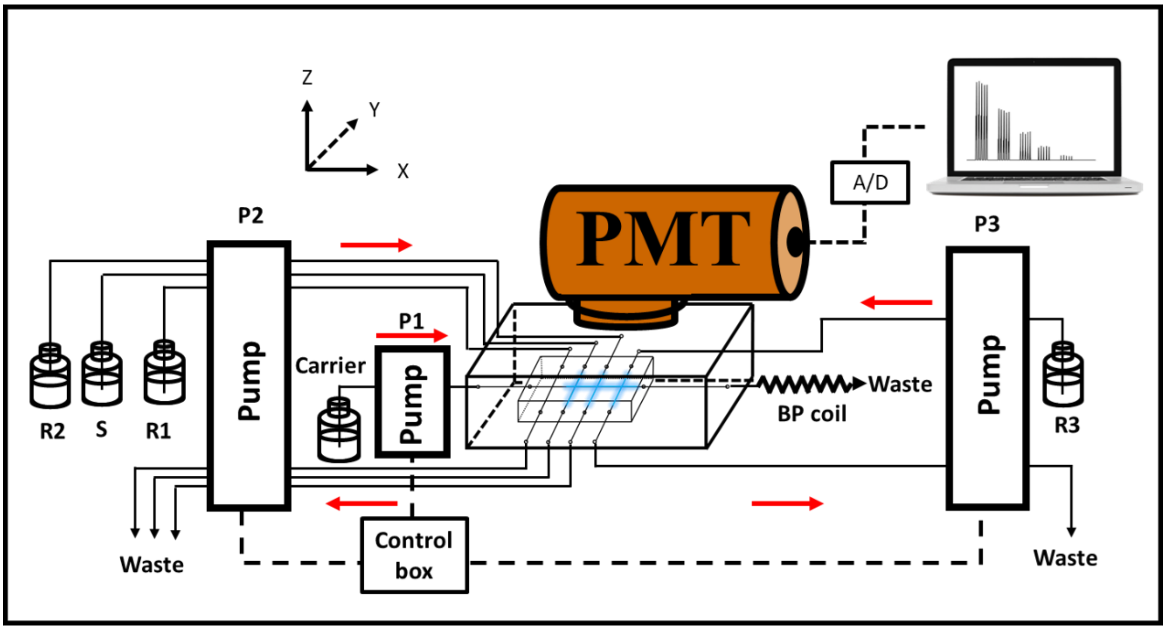

Figure S3. CIA System II for the determination of paracetamol. P1, P2, P3 are peristaltic pumps. BP coil: PTFE tube (0.75 mm. i.d., 1,000 mm). A/D: analog to digital converter. C: carrier solution (1.0 % (*w*/*w*)) H_2O_2 in 0.05 mol L^{-1} phosphate buffer pH 11.5. S: standard paracetamol or sample. R1: 3 mmol L^{-1} luminol in 0.05 mol L^{-1} phosphate buffer pH 11.5. R2: 2.5 mmol L^{-1} $Fe(CN)_6^{3-}$ in 0.05 mol L^{-1} phosphate buffer pH 11.5. R3 is the same solution as the carrier.

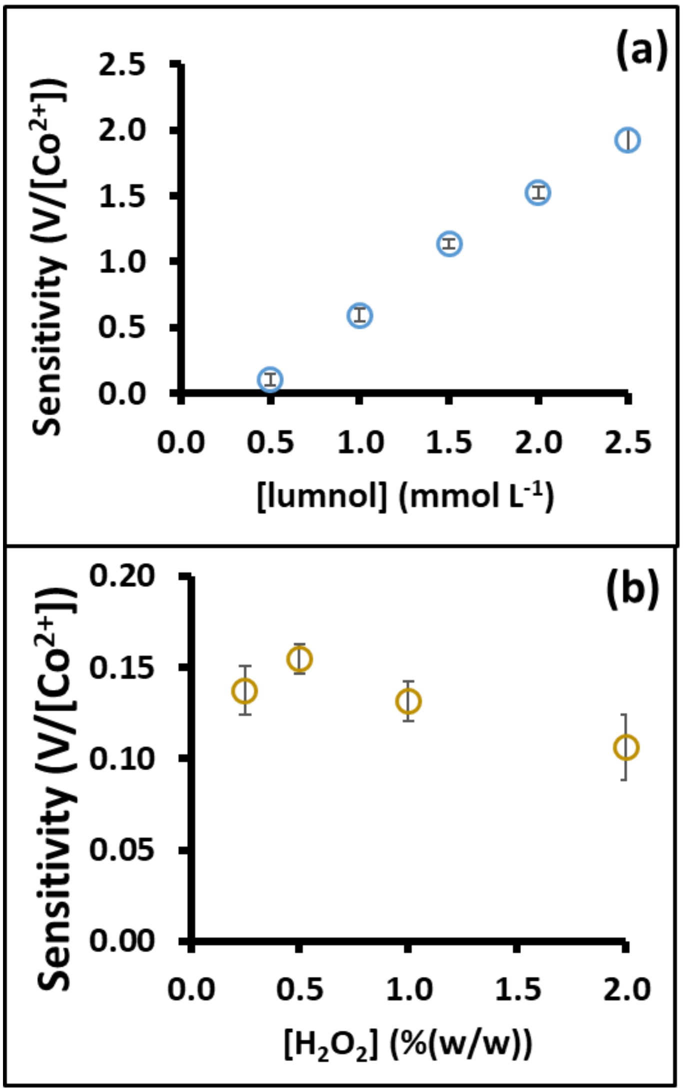

Figure S4. Optimization data of the CIA System I (see Figure. S1) for the determination of Co(II): (**a**) plot of sensitivity vs concentration of luminol in R1, (**b**) plot of sensitivity vs concentration of H_2O_2 (in C, R2 and R3).

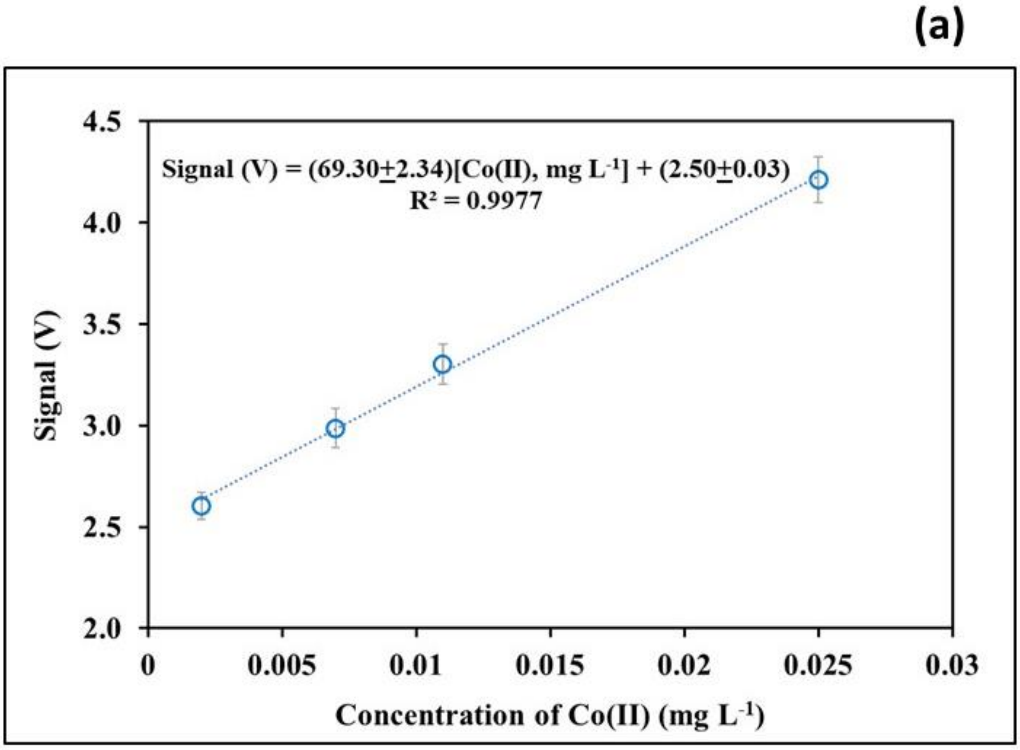

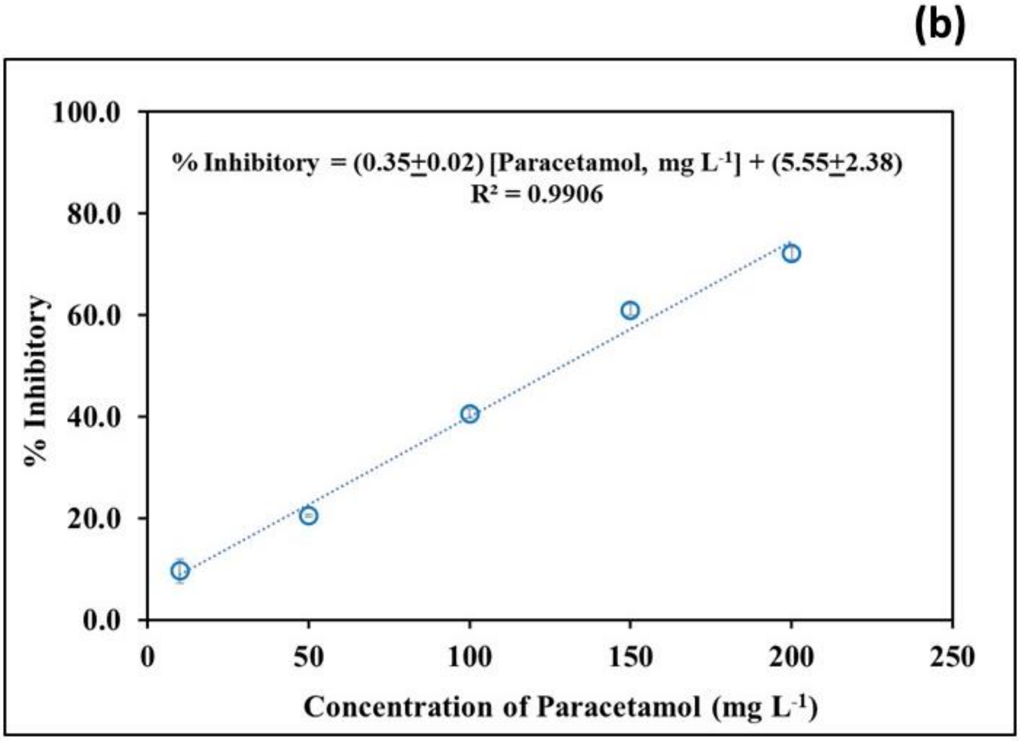

Figure S5. Examples of calibration curves of standard solutions of (**a**) Co(II) (0.5 to 3.5 mg L^{-1}) and (**b**) paracetamol (10 to 200 mg L^{-1}) with their corresponded linear calibration equations.

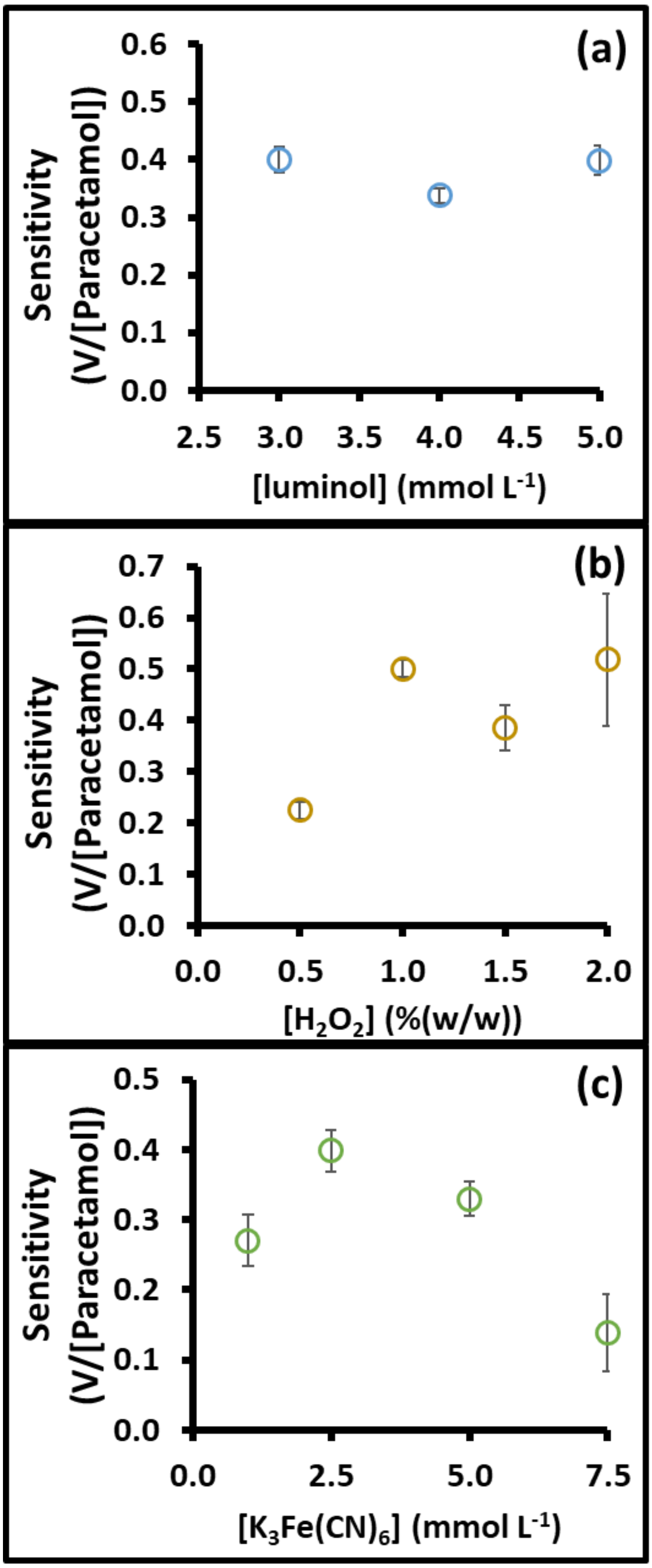

Figure S6. Optimization data of the CIA System II (see Figure S3) for the determination of paracetamol: (**a**) sensitivity vs concentration of luminol in R3, (**b**) sensitivity vs concentration of H_2O_2 (in C and R3), and (**c**) sensitivity vs concentration of $K_3Fe(CN)_6$ in R1.

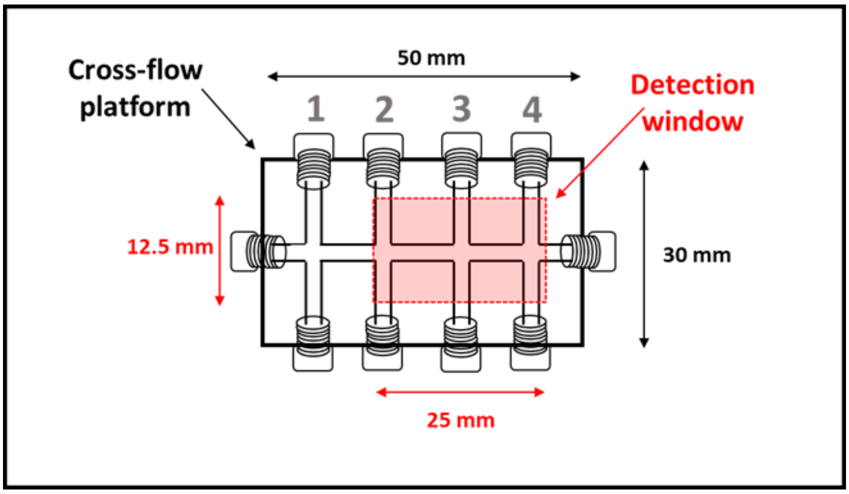

Figure S7. Dimensions of the CIA platform and the approximate detection area by the PMT of the conduits (y-channels No. 2 to No. 4 and the crossing areas).

Table S1. Operation step for "startup procedure" and "routine procedure" employed for CIA system I in Figure S1 for quantitative chemiluminescence analysis of cobalt (II).

Operational Step	P1 (x-Axis Channel)	P2 (y-Axis Channels 1 and 2)	P3 (y-Axis Channels 3 and 4)	Duration Time (s)
Startup Procedure				
1. Filling the conduit with all solutions using x- and y- directional flows	On	On	On	30
2. Loading of the H_2O_2 buffer solution (*via* C, R2 and R3)	On	Off	On	30
3. Loading of sample, luminol solution (R1) and H_2O_2 buffer solution (C)	On	On	Off	10
4. Mixing using x-directional flow	On	Off	Off	20
5. Rinsing with H_2O_2 buffer solution (*via* C, R2 and R3)	On	Off	On	30
Routine procedure				
1. Loading of sample, luminol solution (R1) and H_2O_2 buffer solution (C)	On	On	Off	10
2. Mixing using x-directional flow	On	Off	Off	20
3. Rinsing with H_2O_2 buffer solution (via C, R2 and R3)	On	Off	On	30

Table S2. Operation step for "startup procedure" and "routine procedure" employed for CIA system II in Figure S3 for quantitative chemiluminescence analysis of paracetamol.

Operational Step	P1 (x-Axis Channel)	P2 (y-Axis Channels 1, 2 and 3)	P3 (y-Axis Channels 4)	Duration Time (s)
		Startup Procedure		
1. Filling the conduit with all solutions using x- and y- directional flows	On	On	On	30
2. Loading of the H_2O_2 buffer solution (via C and R3)	On	Off	On	30
3. Loading of sample, $Fe(CN)_6{}^{3-}$ solution (R1), luminol solution (R2) and H_2O_2 buffer solution (via C)	On	On	Off	10
4. Mixing using x-directional flow	On	Off	Off	30
5. Rinsing with H_2O_2 buffer solution (via C and R3)	On	Off	On	20
		Routine Procedure		
1. Loading of sample, $Fe(CN)_6{}^{3-}$ solution (R1), luminol solution (R2) and H_2O_2 buffer solution (via C)	On	On	Off	10
2. Mixing using x-directional flow	On	Off	Off	30
3. Rinsing with H_2O_2 buffer solution (via C and R3)	On	Off	On	20
